# Novel methods to confirm successful puncture in endoscopic ultrasound-guided hepaticogastrostomy

**DOI:** 10.1055/a-2521-4987

**Published:** 2025-02-06

**Authors:** Haruo Miwa, Ritsuko Oishi, Shotaro Tsunoda, Yuichi Suzuki, Kazuki Endo, Hiromi Tsuchiya, Shin Maeda

**Affiliations:** 1 26437 Gastroenterological Center, Yokohama City University Medical Center, Yokohama, Japan; 2 Gastroenterology, Yokohama City University Graduate School of Medicine, Yokohama, Japan

Endoscopic ultrasound-guided hepaticogastrostomy (EUS-HGS) is a widely used procedure; however, it is challenging in patients with a nondilated bile duct 1 . Although, the double-wall puncture (Seldinger’s method) can be helpful in such cases ( Fig. 1 ), specific methods to confirm successful puncture have not been established 2 3 . Aspiration of a large amount of bile can lead to bile duct collapse, making guidewire insertion difficult. On the other hand, if contrast agent is injected without recognizing that the puncture has failed, extravasation may occur outside of the bile duct. Herein, we demonstrate the use of two novel methods to confirm successful EUS-HGS puncture ( Video 1 ).

**Fig. 1:**
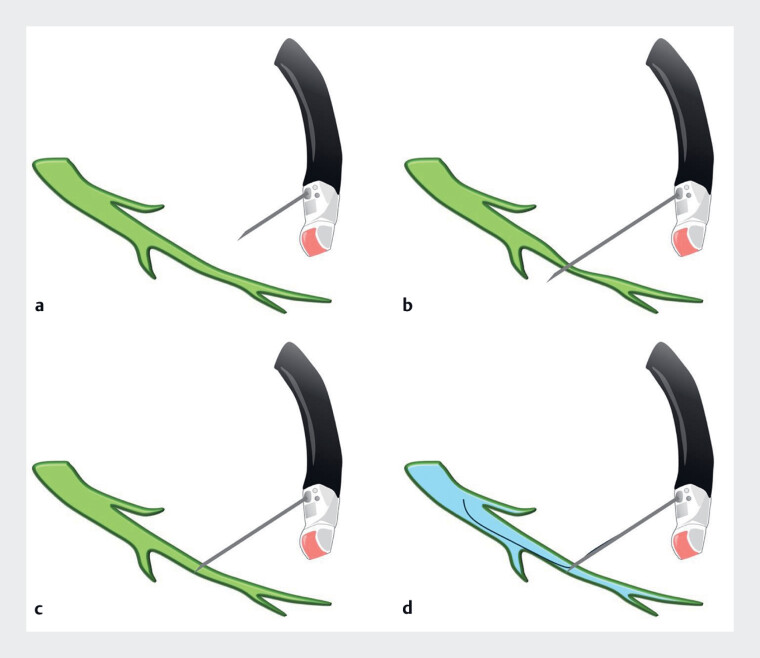
Schema of double-wall puncture showing: **a** a nondilated bile duct; **b** double-wall puncture (puncture of both anterior and posterior wall); **c** pull back of the needle into the bile duct lumen; **d** subsequent contrast injection and guidewire insertion.

Novel methods, the moving bubble sign and Doppler sign, are demonstrated, which can be helpful to confirm successful puncture in endoscopic ultrasound-guided hepaticogastrostomy.Video 1

**Moving bubble sign:** Before puncture, an extension tube and a 10-mL syringe were attached to the end of a 19-gauge needle. The tube and needle were filled with contrast agent, with a small number of air bubbles within it ( Fig. 2 ). After the double-wall puncture, the physician slowly pulled back the needle tip under ultrasound guidance. When the needle tip appeared to return inside the bile duct, the assistant applied slight negative pressure to the syringe. A successful puncture was confirmed when the air bubbles moved smoothly. If the puncture was unsuccessful, the air bubbles remained stationary. Once this sign had been seen, cholangiography was performed successfully.

**Fig. 2:**
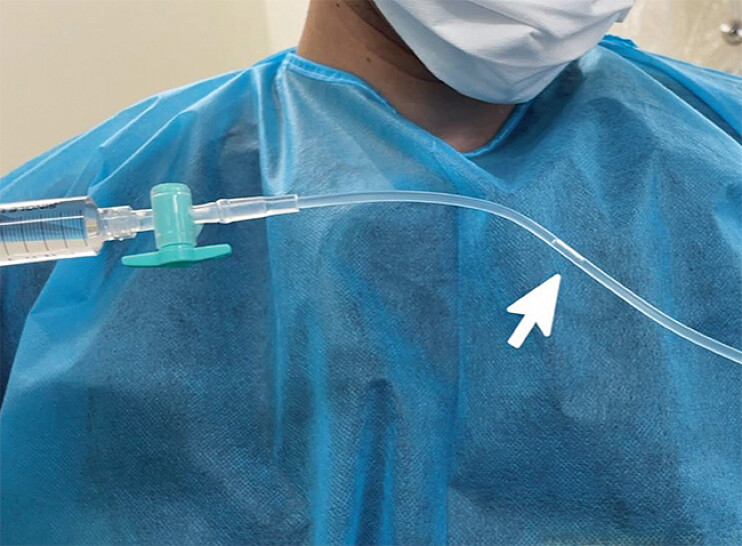
Photograph showing the extension tube filled with contrast agent with a few air bubbles within it that is connected to the end of needle.

**Doppler sign:** During puncture in EUS-HGS, Doppler imaging was used to avoid accidental puncture of the major vessels. When the assistant created negative pressure after successful puncture, the Doppler signal aligned with the needle ( Fig. 3 ). Once this sign had been recognized, cholangiography was successfully performed. The Doppler signal was beneficial to confirm successful puncture when the needle tip visibility was poor.

**Fig. 3:**
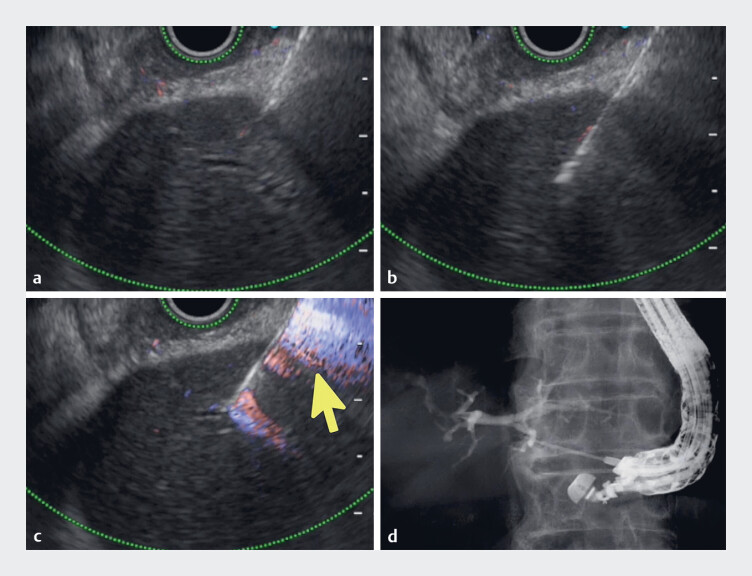
Images of endoscopic ultrasound-guided hepaticogastrostomy being performed in a patient with a nondilated bile duct showing: **a** the nondilated bile duct being punctured with a 19-gauge needle; **b** double-wall puncture being performed; **c** the Doppler signal aligning with the needle when an assistant creates negative pressure; **d** cholangiography being successfully performed.

To the best of our knowledge, this is the first report to describe possible methods for confirmation of successful puncture in EUS-HGS. These methods facilitate cholangiography and guidewire manipulation.

Endoscopy_UCTN_Code_TTT_1AS_2AH

## Notes

Endoscopy E-Videos https://eref.thieme.de/e-videos
*E-Videos* is an open access online section of the journal *Endoscopy* , reporting on interesting cases and new techniques in gastroenterological endoscopy. All papers include a high-quality video and are published with a Creative Commons CC-BY license. Endoscopy E-Videos qualify for HINARI discounts and waivers and eligibility is automatically checked during the submission process. We grant 100% waivers to articles whose corresponding authors are based in Group A countries and 50% waivers to those who are based in Group B countries as classified by Research4Life (see: https://www.research4life.org/access/eligibility/ ). This section has its own submission website at https://mc.manuscriptcentral.com/e-videos .

## References

[1] Ogura T Yamada T Yamada M Contrast-enhanced endoscopic ultrasound-guided access to nondilated bile duct Endoscopy 2019 51 E211 E212 10.1055/a-0885-9722 31049897

[2] Seldinger SI Catheter replacement of the needle in percutaneous arteriography; a new technique Acta Radiol 1953 39 368 376 10.1080/02841850802133386 13057644

[3] Matsubara S Nakagawa K Suda K Practical tips for safe and successful endoscopic ultrasound-guided hepaticogastrostomy: A state-of-the-art technical review J Clin Med 2022 11 1591 10.3390/jcm11061591 35329917 PMC8949311

